# STC-NLSTMNet: An Improved Human Activity Recognition Method Using Convolutional Neural Network with NLSTM from WiFi CSI

**DOI:** 10.3390/s23010356

**Published:** 2022-12-29

**Authors:** Md Shafiqul Islam, Mir Kanon Ara Jannat, Mohammad Nahid Hossain, Woo-Su Kim, Soo-Wook Lee, Sung-Hyun Yang

**Affiliations:** 1 Department of Electronics Engineering, Kwangwoon University, Seoul 01897, Republic of Korea; 2 Graduate School of Knowledge-Based Technology and Energy, Tech University of Korea, Siheung 15073, Republic of Korea; 3 Kwangwoon Academy, Kwangwoon University, Seoul 01897, Republic of Korea

**Keywords:** human activity recognition (HAR), channel state information (CSI), deep learning (DL), nested long short-term memory (NLSTM)

## Abstract

Human activity recognition (HAR) has emerged as a significant area of research due to its numerous possible applications, including ambient assisted living, healthcare, abnormal behaviour detection, etc. Recently, HAR using WiFi channel state information (CSI) has become a predominant and unique approach in indoor environments compared to others (i.e., sensor and vision) due to its privacy-preserving qualities, thereby eliminating the need to carry additional devices and providing flexibility of capture motions in both line-of-sight (LOS) and non-line-of-sight (NLOS) settings. Existing deep learning (DL)-based HAR approaches usually extract either temporal or spatial features and lack adequate means to integrate and utilize the two simultaneously, making it challenging to recognize different activities accurately. Motivated by this, we propose a novel DL-based model named spatio-temporal convolution with nested long short-term memory (STC-NLSTMNet), with the ability to extract spatial and temporal features concurrently and automatically recognize human activity with very high accuracy. The proposed STC-NLSTMNet model is mainly comprised of depthwise separable convolution (DS-Conv) blocks, feature attention module (FAM) and NLSTM. The DS-Conv blocks extract the spatial features from the CSI signal and add feature attention modules (FAM) to draw attention to the most essential features. These robust features are fed into NLSTM as inputs to explore the hidden intrinsic temporal features in CSI signals. The proposed STC-NLSTMNet model is evaluated using two publicly available datasets: Multi-environment and StanWiFi. The experimental results revealed that the STC-NLSTMNet model achieved activity recognition accuracies of 98.20% and 99.88% on Multi-environment and StanWiFi datasets, respectively. Its activity recognition performance is also compared with other existing approaches and our proposed STC-NLSTMNet model significantly improves the activity recognition accuracies by 4% and 1.88%, respectively, compared to the best existing method.

## 1. Introduction

HAR is a process that uses a series of observations of human activity and its surrounding environment to infer the current behaviours and ways of human movement. Recently, research on HAR has drawn more academic and commercial interest because of its numerous applications in different fields, such as smart home [1] (managing different appliances based on human actions for minimizing energy consumption), healthcare [2] (monitoring the health of ageing citizens and disabled people), security [3] (bio-metric or face recognition), and video gaming, etc. Traditional HAR approaches use several sensing technologies: computer vision [4], wearable sensors [5], and radars [6]. Computer vision is a salient sensing technique used to collect information for humans from the surrounding environment and achieve satisfactory results. However, various factors significantly impact activity recognition based on vision, such as difficulty installing cameras everywhere, being affected by light, conflict with the user’s privacy, and influence by environment and occlusion. These limitations greatly affect the output of the vision-based model, which is used in several applications. In wearable-based HAR, data or information are collected using wearable devices and sensors; these devices have seen widespread use and have achieved good results, as well. However, in wearable sensor-based HAR, users must carry electronic devices such as smart bracelets, smart watches, and smartphones all day to collect data, which can be cumbersome, especially for older adults and physically impaired people. Moreover, the devices are costly, and activity recognition is impossible if the person does not carry the necessary devices. On the other hand, radar-based methods use unique devices like universal software radio peripherals to collect data, but these have a short range of coverage.

Several works [7,8,9,10] have demonstrated that WiFi-based HAR has recently gained much attention compared to the approaches mentioned above because of its numerous advantages, including wide coverage, ubiquitous availability, non-intrusiveness, protection of users’ privacy and identities, NLOS communication, and contactless sensing. Literature reveals that existing WiFi-based HAR commonly uses two types of signals: (i) Received Signal Strength Indicator (RSSI) and (ii) CSI. The RSSI signal, which describes coarse-grained information, is frequently utilized in HAR [11] and indoor localization [12]. RSSI signals measure the change in the strength of the received signal. Although RSSI is easy to utilize, it cannot accurately detect changing signals caused by a moving person, and since RSSI measures the strength of the received signal, it becomes inaccurate as the distance between the human action and the receiver increases, thereby affecting the overall accuracy of the system [13]. CSI value is another metric used to identify the various tasks performed by a human. CSI is a fine-grained signal and is used in various domains, such as micro-movement detection [14], pose estimation [15], handwritten recognition [16], and fall detection [17]. CSI easily captures the information of propagating signals between transmit–receive antenna pairs at a specific carrier frequencies. HAR methods using CSI signals are based on the principle that when humans or objects move between the transmitter and receiver antennas, the amplitude and phase of the CSI signals differ from the measurements that are taken when there is no movement. In addition, existing studies have already shown that CSI signals perform better than RSSI signals in a complex environment [18,19]. This is because the amplitude and phase of CSI signals can differentiate between movable and static signal patterns in an indoor environment.

Machine learning (ML) and DL approaches are involved in classifying HAR using WiFi CSI signals. ML-based HAR approaches are highly dependent on manually extracting features from CSI signals using different feature extraction techniques to classify or recognize the human activity. However, manual feature extraction is time-consuming, and new features would be unlikely to manually characterize the data present in the CSI signal’s temporal, frequency and spatial domains. The current DL-based approaches mainly focus on convolutional neural network (CNN) and long-term short memory (LSTM) variant models that can automatically learn features from the input signals instead of manually extracting features. LSTM-based architectures are specifically made for extracting temporal features, whereas CNN-based architectures are made for extracting spatial features. However, the WiFi CSI-based HAR data is time-series data with spatial and temporal features [20]. Therefore, existing DL-based methods for HAR usually use either temporal or spatial features to recognize different human activities. As a result, those CSI-based HAR systems cannot integrate or utilize both features and are unable to extract robust features, which consequently weakens their activity-recognition ability. In this paper, we propose a novel architecture named spatio-temporal convolution with nested LSTM (STC-NLSTMNet), which can capably extract both features simultaneously and automatically recognize human activity with very high accuracy. The STC-NLSTMNet consists of DS-Conv blocks for feature extraction and added FAM to concentrate on the most important spatial features, while NLSTM helps to explore the hidden inherent temporal features to enhance the model’s ability to recognize human activities. Thus, the main contributions of this study can be summarized below:

- We propose a DL-based model (STC-NLSTMNet) which utilizes both features (spatial and temporal) simultaneously and automatically recognizes human activity with very high accuracy.
- In the STC-NLSTMNet model, DS-Conv extracts spatial features, FAM focuses on the most relevant spatial features, and NLSTM explores the hidden inherent temporal features. Thus, the representation capability of the STC-NLSTMNet model is improved to accurately recognize human activity.
- The proposed system is versatile and needs minimal preprocessing of the CSI data or feature engineering, which makes it easy to extend to other activity recognition datasets.

## 2. Related Work

The existing HAR systems are mainly categorized into two groups: wearable-based HAR and non-wearable-based HAR.

### 2.1. Wearable-Based HAR

Khalifa et al. [21] developed a wearable-based HAR system, HARKE, by investigating the effectiveness of the kinetic energy harvesting (KEH) method. They used two datasets: one is public, and another is their collection KEH dataset. They achieved an average of 80–95% accuracy and observed that positioning the sensor or device on the subject’s body affects the overall performance. M. Zubair et al. [22] tried to develop a real-time HAR system by exploring the accelerometer sensor. They used a public dataset and then selected the best relevant features subset and achieved a low recognition rate (90%) because they used a limited set of sensors to collect data. In real-time, HAR needs a high recognition rate, and this can be achieved from a large group of sensors that provide more data and information. In a similar manner, the authors [23,24] made an effort to identify human activity by performing an analysis of the accelerometer data acquired through the use of smartphones. Literature reveals that wearable-based HAR methods have two main limitations: one is need to carry the device, which makes the user uncomfortable, and another is the location of the device on the body, which hampers recognition performance.

### 2.2. Non-Wearable-Based HAR

Non-wearable-based HAR systems can be classified into vision-based, radar-based, ultrasonic sensor-based, and WiFi-based HAR.

#### 2.2.1. Vision-Based HAR

Researchers first attempted HAR using vision-based methods. Vemulapalli et al. [25] presented a low-cost HAR using a depth sensor. They explored the relationship of 3-D geometry between various body parts in a skeletal depiction. They used three public datasets: Florence3D-Action, UTKinect-Action, and MSR-Action3D to test the performance of their approach. The authors claim that their proposed system outperforms various state-of-the-art skeleton-based HAR approaches. Although different systems have focused on gaining high recognition accuracy, most of them have rarely been extended to online recognition. Cheng et al. [26] presented a HAR approach to recognize online and offline human activity by analyzing depth series. They divided the body parts into a set of moving parts and extracted features from each portion, which was used as a set of features. Their proposed model achieved 97.70% accuracy on the MSR Action Pairs dataset. Xia et al. [27] suggested using histograms of the joints’ 3D locations (HOJ3D) as a small depiction of human postures. The collected feature vectors were analyzed using the Hidden Markov Model (HMM), which were then utilized to construct the classification models and achieved 95.0% accuracy. There are some limitations to implementing vision-based systems in a real-time application, such as invasion of the user’s privacy and failure to detect activity if the user is behind an obstacle.

#### 2.2.2. Radar-Based HAR

Karayaneva et al. [28] employed an unsupervised framework based on Doppler radar to recognize daily activities in e-healthcare. They achieved about 80% accuracy. Kim et al. [29] proposed a radar-based HAR approach using DL. They showed that the pro-posed RD-CNN achieved better accuracy on a publicly available radar-based HAR dataset. Franceschini et al. [30] analyzed the doppler signature of a radar sensor and proposed a low-cost hand gesture recognition approach using CNN. They achieved 97% accuracy. The available radar data is limited, which is behind the real-time scenario. The procedure of labelling and collecting a large number of radar data points requires a significant amount of manpower and investment.

#### 2.2.3. Ultrasonic Sensor-Based HAR

Arindam et al. [31] explored the use of ultrasonic sensors to detect different human activities (sitting, standing, and falling). They employed three ML classifiers and, for all classifiers, achieved an accuracy of 81–90%. Hori et al. [32] presented a system that would utilize an ultrasonic sensor network to monitor the actions of older people to detect and avoid accidents such as falls. Xiong et al. [33] presented a technique to identify a person’s presence utilizing a pyroelectric infrared sensor.

#### 2.2.4. WiFi-Based HAR

The current WiFi-based research work can be divided into RSSI-based and CSI-based approaches. The RSSI format describes coarse-grained data about a communication channel. RSSI-based HAR systems depend on changes in received signal strength induced by human actions. Sigg et al. [34] proposed a device-free HAR system where a mobile phone was used to collect RSSI signals. After data collection, they extracted features and applied a feature selection method to select the best features. Their system achieved an accuracy of 52% for the recognition of eleven gestures and 72% for the recognition of four gestures. Gu et al. [35] presented an online HAR system that explored WiFi RSSI fingerprints of different human activities. Y. Gu et al. [36] proposed a novel recognition framework using an RSSI signal. They devised a unique fusion approach that combines the k-NN algorithm with a classification tree and achieved an accuracy of 92.58%. Their framework achieved accuracy of 92.58%. Sigg et al. [37] presented a hardware device USRP, to collect RSSI signals from WiFi devices. They utilized RSSI signals to identify four activities: standing, walking, lying down, and crawling, and achieved more than 80% accuracy. Although RSSI-based HAR has achieved promising results, it suffers from some problems. RSSI does not work well on complex activities. RSSI only provides coarse information about channel fluctuations; multipath effects and noise frequently influence it.

In contrast to RSSI, CSI has recently been used to localize and classify human activity because it provides a more fine-grained presentation of the wireless link. An indoor localization and HAR system were proposed by Wang et al. [38]. The authors created a dataset for six different types of human activities and proposed a multi-task 1D CNN with a ResNet as a basic architecture, and their proposed model achieved an accuracy of 95.68%. Yang et al. [39] developed a framework for HAR using a WiFi CSI signal. They first proposed an algorithm for automatically selecting an antenna based on its sensitivity to various activities. After that, two signal enhancement techniques were applied to detect active and inactive signals, respectively. They employed three ML classifiers and a CNN model to evaluate and achieved an average accuracy of 96.82%. The HAR dataset used was collected in only one environment and suffered from insufficient performance. Damodaran et al. [40] proposed a HAR system able to classify four types of human activity using CSI signals. They gathered datasets from two environments that took place in an indoor setting: LOS and NLOS. From the experimental results, they found that LSTM models achieve more accuracy (97.33%) than SVM with less processing data. They collected only four human activities, which is far less than the real environment. Yousefi et al. [41] developed a dataset containing six different human activity types. They used principle component analysis (PCA) to denoise their developed dataset. To evaluate the performance of the proposed dataset, they used different types of models, namely the HMM, LSTM, and a random forest. From the experimental results, the authors observed that the LSTM model gives the highest accuracy (90.5%) among all models. Their method required more computational cost and failed to achieve better performance. Based on a WiFi CSI signal, Santosh et al. [42] suggested an altered version of the Inception Time network architecture, which they referred to as CSITime for HAR. They evaluated their system with three different datasets, namely the ARIL dataset, the StanWiFi dataset, and the SignFi dataset, and they obtained an accuracy of 98.20%, 98%, and 95.42%, respectively, using these datasets. CSITime showed good performance regarding ARIL and the StanWiFi dataset but failed regarding the SignFi dataset. Wang et al. [43] presented a CSI-based CARM theory in which they claimed that their proposed approaches achieved activity recognition accuracy of 96%, but this result is insufficient. Chen et al. [19] proposed a bi-directional LSTM based on attention called ABLSTM. Their proposed ABLSTM model achieved similar accuracy (97.3%) on the public dataset and the dataset collected in the meeting room. Yan et al. [44] introduced a CSI-based HAR system that could differentiate between activities based on the relationship between the amplitude of body movement and the magnitude of the movement. They came up with something called the Adaptive Activity Cutting Algorithm (AACA), which helped them achieve an accuracy rate of 94.20% on average, which is poor performance compared to HAR-based methods. Muaaz et al. [45] proposed a HAR method and used their own collected dataset. They produced spectrogram images by employing STFT as an input to CNN. The authors reported that their proposed system achieved an accuracy of 97.78%. Jin et al. [46] proposed a dense neural network with bi-directional LSTM (Dense-LSTM) using CSI signals. Their method achieved an accuracy of about 90% while utilizing modest amounts of CSI data but did not address how using a large dataset affected their performance. Shang et al. [47] pro-posed a DL model combining LSTM-CNN using a WiFi CSI signal. Their framework showed an average result of 94.14% on the public dataset. Pritam et al. [48] proposed a DL architecture LSTM to extract features automatically. They achieved the best accuracy of 92.29% when using CSI amplitude-phase together, but accuracy decreased while using only phase signal. Alsaify et al. [49] presented a multi-environment HAR system based on CSI signal. They extracted several statistical features from their own collected dataset and selected the best feature subset. They achieved an accuracy of 91.27% using the SVM classifier. Their approach needs a great deal of preprocessing and the performance is relatively low.

From the above discussion, it is clear that numerous researchers have worked on recognizing human activity using various approaches, including feature extraction, ML, LSTM, and CNN. However, WiFi CSI-based HAR data contains both spatial and temporal features, necessitating the use of a robust model that can learn both aspects of human activity. Therefore, in this work, we propose a model (STC-NLSTMNet) that can capture spatial-temporal features for effective HAR with fewer parameters and high accuracy.

## 3. Background of CSI

In wireless communication, CSI represents the channel properties of any communication links. These properties refer to the signal propagation caused by the object moving between the transmit–receiver antenna pair and also the effects of scattering, reflection, refraction, etc. CSI can represent how a signal changes (such as amplitude attenuation, phase information, and time delay) between the transmitted and received signals. Wireless communication systems are improving using MIMO, which comprises multiple pair of antennas that can send and receive signals. Using OFDM, a MIMO system divides the channel’s available bandwidth into a number of orthogonal subcarrier frequencies that are transmitted simultaneously. The communication system can be modelled by the following mathematical Equation (1)

(1) $$y_{i}=H_{i}x_{i}+V,\;\;\;i\;=\;1,\;2,\;3\ldots ,\;N$$

$H_{i}$ indicates complex-valued matrix of the *i*th OFDM subcarrier, ***V*** represent noise, ***N*** represent the number OFDM subcarrier; $y_{i}\in \;{\mathbb{R}}^{N_{R_{a}}}$ and $x_{i}\in \;{\mathbb{R}}^{N_{T_{a}}}$ are the *i*th transmitted and received signal, respectively. The basic structure of $H_{i}$ is given below

(2) $$H_{i}=\left[\begin{array}{ccc} h_{i}^{T_{\;a_{1}}R_{a_{1}}} & \cdots & h_{i}^{T_{\;a_{j}}R_{a_{1}}}\\ \vdots & \ddots & \vdots \\ h_{i}^{T_{\;a_{j}}R_{a_{1}}} & \cdots & h_{i}^{T_{\;a_{j}}R_{a_{k}}} \end{array}\right]$$

Here, $h_{i}^{T_{\;a_{p}}R_{a_{q}}}$ represents the complex matrix of CSI of *i*th OFDM subcarrier between *p*th transmitted antenna and qth receiving antenna. $h_{i}^{T_{\;a_{p}}R_{a_{q}}}$ can be expressed as:

$$h_{i}^{T_{\;a_{p}}R_{a_{q}}}=|h_{i}^{T_{\;a_{p}}R_{a_{q}}}|\;e{∠}^{h_{i}^{T_{\;a_{p}}R_{a_{q}}}}$$

where |$h_{i}^{T_{\;a_{p}}R_{a_{q}}}$| and ${∠}^{h_{i}^{T_{\;a_{p}}R_{a_{q}}}}$ represent the amplitude and phase value of CSI, respectively.

Amplitude and phase information are included in the CSI measurements. In this study, we utilized the amplitude of the CSI for HAR, leaving the phase information for future research.

## 4. Dataset

We have conducted our experiment on two online available datasets, one is Multi-environment and another is StanWiFi. The detailed description of both datasets is provided below:

### 4.1. Multi-Environment

The Multi-environment dataset [49,50] was collected in three different environments i.e., E1, E2, and E3, where E1 and E2 were LOS and E3 was NLOS. In the same LOS scenario, E1 was an office room and E2 was a hall room, with only the distance difference between the transmitter and the receiver. In the office room, the receiver was 3.7 m away from the transmitter, while in the hall room, the distance was 7.6 m. In contrast, the E3 had a thin wall (8 cm) between the transmitter and the receiver, which is diverse compared to other CSI-based HAR datasets. They used two computers equipped with CSI tools where one computer acted as a transmitter with one antenna and the other computer was assigned as a receiver with three antennas. Thirty persons were asked to perform five pre-defined experiments in every environment, repeated 20 times. Thus, the total number of trials in the dataset was 3000 (30 persons × 5 experiments × 20 trials). Each pre-defined experiment was further divided into several activities, and we used those activities in our study to evaluate our method. For example, Sit-down and Stand-up was further divided into four activities: Sitting still on a chair, standing up, standing still, and then sitting down on the chair. The sampling rate of this dataset is 320 packets per second, which is higher than other datasets that have only 20 or 30 packets per second. The activities and designations we used next in this study are provided in Table 1.

### 4.2. StanWiFi Dataset

The other dataset, named StanWiFi, with which we conducted our experiments is provided in [41]. Six subjects were asked to perform six daily activities such as sitting down, standing up, lying down, running, walking, and falling in an indoor circumstance where each action was repeated 20 times. During data collection, a Wi-Fi router served as a transmitter with one antenna, and a computer equipped with a NIC 5300 served as a receiver with three antennas. The transmitter and receiver were located 3 m apart in the LOS situation. Each activity was performed for a period of 20 s, while at the start and the end of each activity, the person remained stationary. The sampling frequency of the dataset was 1000 Hz. Our proposed model is evaluated on these six activities. The data distribution of the StanWiFi dataset is given in Table 2.

## 5. Proposed Methodology

**Figure 1:**
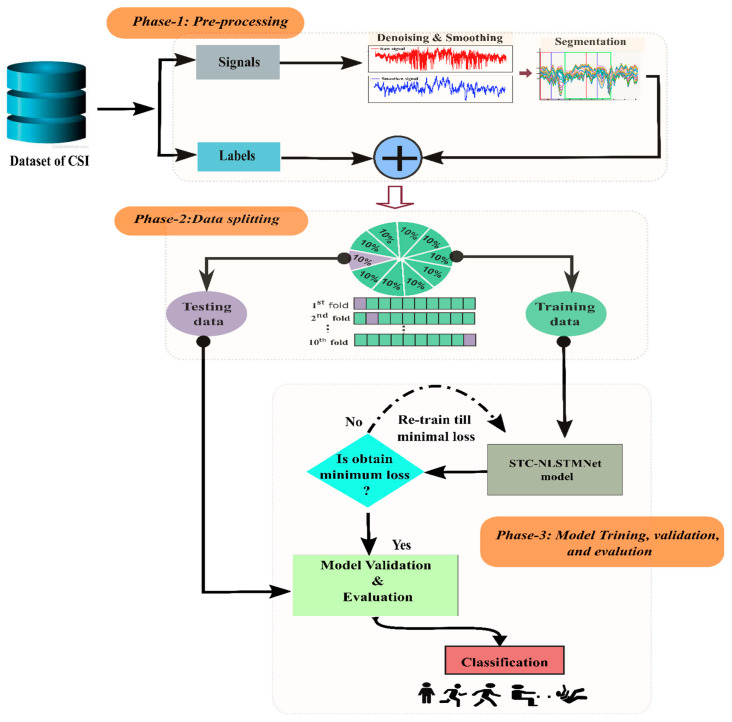
Block diagram of procedural steps of the proposed STC-NLSTMNet model to recognize human activity.

The methodological steps in our proposed system to recognize human activity are shown in a block diagram in Figure 1. The proposed system consists of three main stages: (i) Raw CSI data preprocessing. (ii) Splitting the processed data into ten (10) folds for training and testing, respectively. (iii) Proposed (STC-NLSTMNet) model train, validation and evaluation.

### 5.1. Data Preprocessing

**Figure 2:**
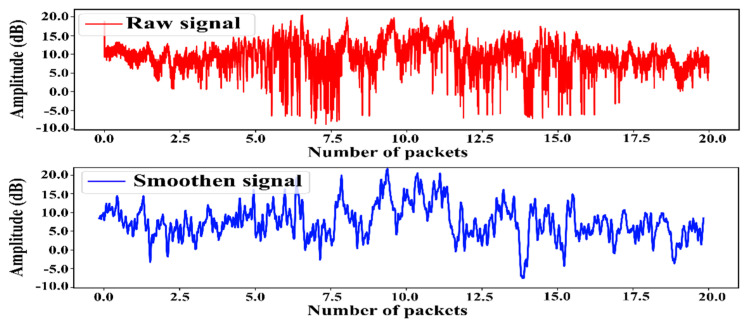
Visualization of the raw and smoothen CSI signal for Run activity.

Noise is created in the raw amplitude of the CSI signal due to high-frequency environmental noise and multipath effects during propagation, reducing the final recognition result. Therefore, before feeding to the model, it is vital to eliminate this high-frequency noise. Hence, in this work, we used a Butterworth filter to denoise the raw signal and retain full information of activity. Gaussian smoothing was then applied to attenuate the minor peaks. Figure 2 shows the raw and smoothed CSI signals for the Run activity. The duration of the recorded CSI signals can vary, and processing a CSI signal with a longer length requires both more time and computational power. We experimented with various window lengths and observed that using a window length of 512 yields the highest recognition accuracy. Therefore, we made use of a sliding window with a size of 512 and a stride of 128 on both datasets.

### 5.2. STC-NLSTMNet

**Figure 3:**
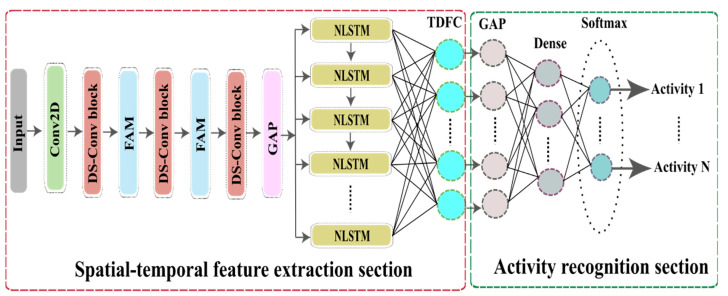
The schematic diagram of the STC-NLSTMNet model.

Figure 3 illustrates the two primary sections that make up the proposed STC-NLSTMNet model: spatial-temporal feature extraction (STFE) and recognition. The STFE section has one 2D CNN, three DS-Conv blocks, two FAM, and NLSTM. 2D CNN and DS-Conv blocks extract the spatial features from CSI and FAM helps to focus the most significant spatial features. Then, these robust features are fed into NLSTM as inputs to capture the hidden intrinsic temporal features of CSI signals. The NLSTM is not the same as either the standard LSTM or the stacked LSTM. When compared to stacked LSTM, which involves stacking a number of LSTM one on top of the other, with the output of each layer serving as the input to the subsequent layer, the NLSTM enhances the depth of LSTM through nesting to select and use information from CSI signals in a more efficient manner. In NLSTM, the internal memory cells are able to retain and process long-term features of CSI signals, while the external memory cells are responsible for data selection and processing. Therefore, The NLSTM can extract long-term temporal features of CSI signals more stable and effectively. A time-distributed fully connected (TDFC) layer is utilized to acquire the output characteristics of the NLSTM layer at each of its time steps. This TDFC layer uses the outputs of all time steps of NLSTM rather than only using the most recent time steps output. On the contrary, in the recognition section, the global average pooling (GAP), fully connected (FC), and softmax layers are used. As a result, the STC-NLSTMNet model synergistically integrates DS-Conv, FAM, and NLSTM to simultaneously extract both features and automatically recognize human activities with very high accuracy. Table 3 contains a tabular representation of a summary of the proposed STC-NLSTMNet model.

#### 5.2.1. Depthwise Separable Convolutional Block (DS-Conv)

DS-Conv [51] is a factorized form of the standard convolution *S_CNN_*. *S_CNN_* applies a filter with a convolution operation on input in one step to produce an output which requires large memory as well as computational complexity. The DS-Conv approach can be an alternate solution in this regard. DS-Conv significantly reduces the computational complexity of the method in that it splits the whole S_*CNN*_ method in two ways, namely, depthwise convolution (*D_Conv_*), and pointwise convolution (*P_Con_*_v_). Initially, a filter is applied in each channel separately to learn the spatial features utilizing in *D_Conv_*, and finally, all the features are combined depthwise in *P_Conv_*.

Considering an input space of $I_{H}\times I_{W}\times M$ which applied an S_CNN_ to produce an output of $O_{H}\times O_{W}\times N$, where subscript H and W indicate the height and width of the input and output data, and M and N represent the input and output channel/depth. If the kernel or filter K of shape $K_{H}\times K_{W}\times M\times N$ where subscript H and W indicate the size of kernel or filter height and width of *S_CNN_* layer, then the output and computational complexity for any *S_CNN_*:

(3) $$O{\left(S_{CNN}\right)}_{k,l,n\;}=\underset{i,j,m}{\sum }K{\left(S_{CNN}\right)}_{i,j,m,n}\cdot I_{k+i-1,l+j-1,m}$$

(4) $$C_{SCNN}=K_{H}\cdot K_{w}\cdot M\cdot N\cdot O_{H}\cdot O_{W}$$

*D_Conv_* applies a single convolution filter or kernel to each input channel, while *P_Conv_* performs 1×1 convolution to combine the *D_Conv_* outputs in order to create the same output as *S_CNN_*.The output and computational complexity for D_Conv_ yields

(5) $${\left(D_{Conv}\right)}_{k,l,m\;}=\underset{i,j}{\sum }K{\left(D_{Conv}\right)}_{i,j,m}\cdot I_{k+i-1,l+j-1,m}$$

**Figure 4:**
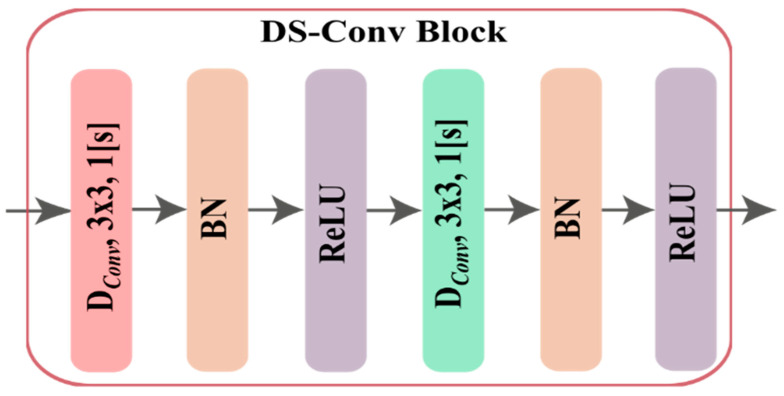
Block diagram of DS-Conv block.

Every DS-Conv block is made up of *D_Conv_* with 3×3 kernels of stride 1, followed by batch normalization (BN), ReLU, and *P_Conv_* with 1×1 kernels of stride 1, followed by BN, ReLU. The schematic diagram of DS-Conv is presented in Figure 4.

#### 5.2.2. Feature Attention Module (FAM)

The crucial shortcoming of CNN is that rather than relating local features, it instead focuses on extracting local features from input data. As a remedy, several works address the attention concept either spatially or in channel, which greatly contributes to improving the performance of CNNs. The FAM utilizes average pooling and max pooling to determine how the two neighbours’ various descriptive local features relate to one another. To motivate with the usefulness of FAM, we utilized FAM block for extracting fine-grained features. The mechanism of FAM is as follows: initially, spatial features are extracted from the features map received from the earlier layer through average-pooling and max-pooling operations. After that a concatenation operation is performed to get the resultant features. Finally, a 2D convolution operation with a sigmoid activation function is applied to generate the feature attention map.

**Figure 5:**
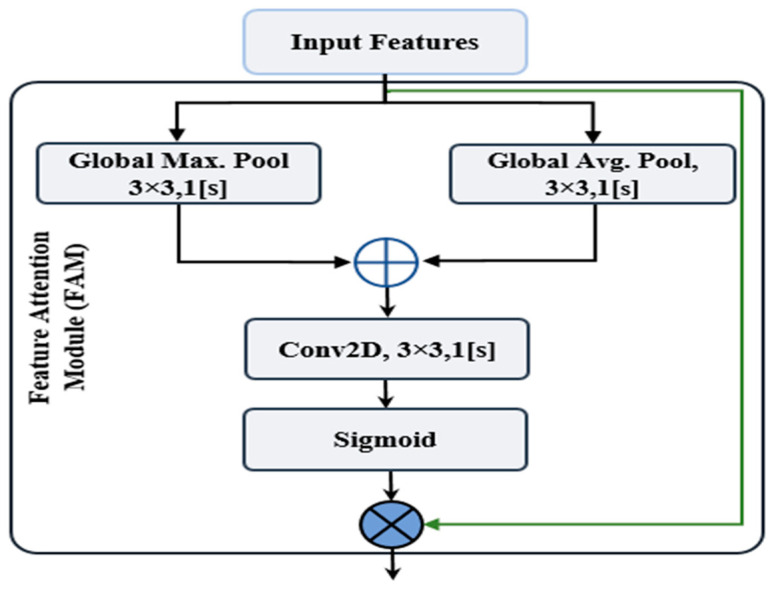
The FAM’s schematic diagram.

As shown in Figure 5, the input features (X) undergo global max pooling and global average pooling operations in parallel: $x_{max}\in X$ and $x_{avg}\in$ X, where $x_{max}$ and $x_{avg}$ presents max pooling and average pooling, respectively. A 2D convolution operation is employed after an element-wise concatenation with a 3×3 single kernel and a stride size of 1. The output feature map FAM(x) is linearized by the sigmoid activation function (***x***). To get final features $\tilde{x}$, using element wise multiplication, FAM(x) is also matched with input features (X).

$$x_{avg}=AvgPool\left(x\right),$$

$$x_{max}=MaxPool\left(x\right),$$

$$FAM\left(x\right)=\sigma \left(f^{3\times 3}Concat\left(\left[x_{avg}\right];\left[x_{max}\right]\right)\right),$$

$$\tilde{x}=x⊙FAM\left(x\right),$$

where σ indicates the sigmoid activation function, $f^{3\times 3}$ is the 3×3 single kernel, Concat() is the concatenation operator, and ⊙ is element-wise multiplication.

#### 5.2.3. Nested Long Short-Term Memory (NLSTM)

**Figure 6:**
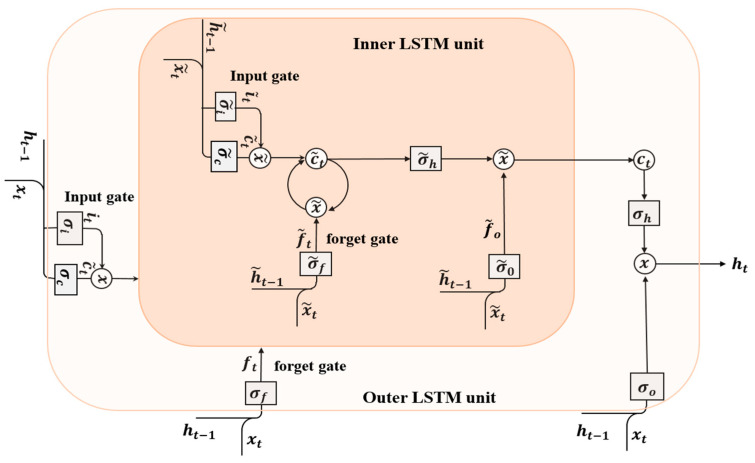
Functional Block diagram of NLSTM.

NLSTM [52] is a factor of RNN model with multiple levels of memory. In contrast to staked LSTM, NLSTM is nested together LSTM units. NLSTM is used due to its features of good robustness and generalization power in different domains. NLSTM explores the inherent temporal features in the inputs to capture high-level features. The operation of a memory cell in an NLSTM is mimicked by a standard LSTM cell. The function of the output gate in LSTM is to encode the memory, which is not even relevant at the current time-step, and to remember it for future use. Similarly, NLSTM utilizes adopt it to create a temporal hierarchy of memory and can access the inner memory immediately, but uses a selection approach for longer-term information, which is only relevant occasionally. The schematic diagram of NLSTM is shown in Figure 6.

The mathematical relationship between gates and cell input of the inner LSTM with the outer LSTM are as follows:

(6) $${\tilde{i}}_{t}={\tilde{\sigma }}_{i}\left({\tilde{x}}_{t}{\tilde{W}}_{xi}+{\tilde{h}}_{t-1}{\tilde{W}}_{hi}+{\tilde{b}}_{i}\right),$$

(7) $${\tilde{f}}_{t}={\tilde{\sigma }}_{f}\left({\tilde{x}}_{t}{\tilde{W}}_{xf}+{\tilde{h}}_{t-1}{\tilde{W}}_{hf}+{\tilde{b}}_{f}\right)$$

(8) $${\tilde{o}}_{t}={\tilde{\sigma }}_{c}\left({\tilde{x}}_{t}{\tilde{W}}_{xo}+{\tilde{h}}_{t-1}{\tilde{W}}_{ho}+{\tilde{b}}_{o}\right)$$

(9) $${\tilde{c}}_{t}={\tilde{f}}_{t}⊙{\tilde{c}}_{t-1}+{\tilde{i}}_{t}⊙{\tilde{\sigma }}_{c}\left({\tilde{x}}_{t}{\tilde{W}}_{xc}+{\tilde{h}}_{t-1}{\tilde{W}}_{hc}+{\tilde{b}}_{c}\right)$$

(10) $${\tilde{h}}_{t}={\tilde{o}}_{t}⊙{\tilde{\sigma }}_{c}({\tilde{c}}_{t})$$

(11) $${\tilde{x}}_{t}=i_{t}⊙\sigma_{c}\left(x_{t}W_{xc}+h_{t-1}W_{hc}+b_{c}\right)$$

(12) $${\tilde{h}}_{t-1}=f_{t}⊙{\tilde{c}}_{t-1}$$

where ${\tilde{i}}_{t}$, ${\tilde{f}}_{t}$, and ${\tilde{o}}_{t}$ are the states of the three gates of inner LSTM unit; ${\tilde{c}}_{t}$ represents the cell input state of inner LSTM unit; ${\tilde{h}}_{t}$, ${\tilde{x}}_{t}$, and ${\tilde{h}}_{t-1}$ are the inputs of the inner LSTM unit; $x_{t}$ and $h_{t-1}$ are the inputs of the outer LSTM unit; σ denotes the activation function in the LSTM unit; ${\tilde{W}}_{xf},{\tilde{W}}_{xi},{\tilde{W}}_{xo}$ and ${\tilde{W}}_{xc}$ denotes the respective weight vector between ${\tilde{x}}_{t}$ to all gates and the cell input; ${\tilde{W}}_{hi},{\tilde{W}}_{hf},{\tilde{W}}_{ho}$ and ${\tilde{W}}_{hc}$ denotes the respective weight vector between ${\tilde{h}}_{t-1}$ to all gates and the cell input; ${\tilde{b}}_{i},{\tilde{b}}_{f},{\tilde{b}}_{o}$ and ${\tilde{b}}_{c}$ denote the biases of the all the gates and the cell input; and ⊙ means the scalar product of two vectors. The cell state of the outer LSTM

$$c_{t}={\tilde{h}}_{t},$$

where ${\tilde{h}}_{t}$ is the output of the inner LSTM unit.

### 5.3. Training of STC-NLSTMNet Model

How well a model will perform and be trained depends on having a sufficient amount of data variation and the selection of appropriate hyperparameters. The choice of hyperparameters means selecting the number of epochs, batch size, activation function, and learning rate. Hyperparameter selection is used in the training phase, while performance evaluation involves using the validation set. Therefore, we adopt the following hyperparameter during the training phase: epochs = 100, batch size = 128, and learning rate, α = 1 × 10^−3^. A significant issue in the training phase is overfitting. Here, we apply the Adam optimizer to minimize the overfitting hazard and optimize the categorical cross-entropy function. Besides, the proposed FAM only selects the most important features, which also lowers the probability of over-fitting. In this work, we adopt the early stopping technique [53], which stops the current training epoch if it is noticed that the validation accuracy has not increased for six successive epochs. The model that has the least amount of lost value is the one that is kept as the best model.

This study used two CSI-based public datasets (StanWiFi and Multi-environment) to validate the proposed method’s performance. This study exploited the 10-fold cross-validation (CV) technique to examine the stability and dependability of the proposed STC-NLSTMNet model. The 10-fold CV technique randomly divided the whole dataset into 10 non-overlapping subsets, where nine subsets are picked for training purposes and the remaining subset is for testing. This process is repeated 10 times, and the final accuracy of recognition is the average of those 10 times.

We employ widely known performance evaluation criteria, namely, accuracy, F1-score, precision, and recall, to measure the recognition performance of the proposed system. Accuracy measures the total percentage of the accurate recognition rate of the classifier. The F1 score is the weighted mean of recall and precision. The evaluation metrics are as follows:

(13) $$\mathrm{Accuracy}=\frac{\mathrm{TP}+\mathrm{TN}}{\mathrm{TP}+\mathrm{FP}+\mathrm{FN}+\mathrm{TN}}\times 100$$

(14) $$\mathrm{Precision}=\frac{\mathrm{TP}}{\mathrm{TP}+\mathrm{FP}}\times 100$$

(15) $$\mathrm{Recall}=\frac{\mathrm{TP}}{\mathrm{TP}+\mathrm{FN}}\times 100$$

(16) $$F1-\mathrm{score}=\frac{2\times \mathrm{precision}\times \mathrm{recall}}{\mathrm{precision}+\mathrm{recall}}\times 100$$

Here, TP classifies the positive class as positive, FP classifies the positive class as negative, TN classifies the negative class as negative, and FN classifies the negative class as positive.

## 6. Results and Discussion

In this section, we present the experimental results obtained using the proposed STC-NLTMNet model on two separate datasets: StanWiFi and Multi-environment. In addition to the performance result of the proposed STC-NLSTMNet model, we also conducted an analysis of a comparative study of the proposed STC-NLSTMNet model with other state-of-the-art models on the two datasets.

### 6.1. Experimental Results on StanWiFi Dataset

Table 4 shows the performance result of the proposed STC-NLSTMNet model of HAR, demonstrating superior classification performance on the StanWiFi dataset. It can be observed from Table 4 that our STC-NLSTMNet model achieved an average accuracy of 99.88%, a precision of 99.72%, a recall of 99.73%, and an F1-score of 99.72%, respectively, for all human activities. Further analysis of Table 4 reveals that among the ten folds, the 7th fold has the maximum performance of 100% accuracy, and other remaining folds achieved more than 99% accuracy, demonstrating that our STC-NLSTMNet model has exceptional classification performance for HAR tasks.

**Figure 7:**
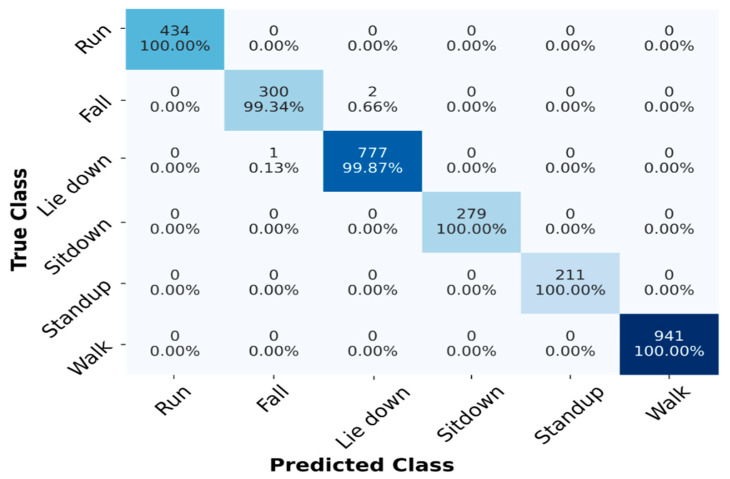
Confusion matrix of the proposed model on StanWiFi dataset.

The confusion matrix of the proposed STC-NLSTMNet model on the StanWiFi dataset is shown in Figure 7, where the main diagonal presents the individual recognition accuracy of human activities. From Figure 7, we can observe that the activities, i.e., Run, Sitdown, Standup, and Walk, achieve 100% accuracy, which demonstrates that the proposed STC-NLSTMNet model can capture both spatial and temporal features inside of the WiFi CSI signal. Although misclassification arises between Lie down and Fall, the possible reason might be that in Fall activities, subjects suddenly fall down and remain steady for some time, which shows a similar pattern as Lie down activities.

### 6.2. Experimental Result on Multi-Enviroment Dataset

The experimental results of the STC-NLSTMNet model for HAR on both the LOS and NLOS are shown in Table 5 and Table 6. We evaluated the performance on LOS scenarios, namely E1 (Office room) and E2 (Hall room), and an NLOS scenario, namely (E3). In Table 5, we can see that the STC-NLSTMNet model obtains an average accuracy of 98.20%, precision of 98.10%, recall of 98.08%, and F1-score of 98.09% with respect to the E1 scenario. On the other hand, the proposed model achieves an average accuracy of 96.65%, precision of 96.54%, recall of 96.41%, and F1-score of 96.48% with respect to the E2 scenario. The proposed STC-NLSTMNet model accuracy dropped about 2% when we experimented with E2. This reduction of recognition accuracy may be because the distance (7.6 m) between the transmitter and receiver antennas at E2 is more than the distance (3.7 m) at E1. Consequently, problems associated with signal fading and multi-path interference are more prevalent in the E2 than in the E1. Although the proposed model improves the E1 and E2 by 4% and 7% accuracy, respectively, in the existing state-of-art works (see in Table 7).

Table 6 shows that the proposed STC-NLSTMNet model achieved an average accuracy of 94.68%, precision of 94.57%, recall of 94.55% and F1-score of 94.56%, respectively, for all activities. Compared to scenarios LOS (E1 and E2), our proposed model shows lower performance in scenario NLOS (E3). The possible reason would be the thin wall (8 cm) between the transmitter and receiver antennas and the distance (5.4 m) between them. Those factors cause more multi-propagation (reflection, refraction, and fading) of the signals, which affects the activity-recognition accuracy.

**Figure 8:**
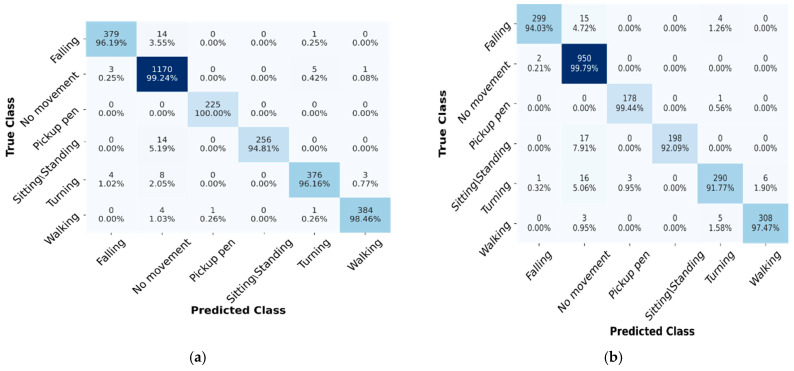
Confusion Matrix of the proposed STC-NLSTMNet model on LOS. (**a**) E1. (**b**) E2.

Figure 8a,b show the confusion matrix of the proposed STC-NLSTMNet model for the LOS scenario (E1 and E2). In the E1 scenario, the activities, i.e., Falling, No movement, Pickup pen, Turning, and Walking, have over 96% recognition accuracy, whereas only the Sitting/Standing activity shows less (about 94%) performance. On the other hand, the activities, i.e., Falling, No movement, Pickup pen, Sitting/Standing and Walking, show recognition accuracy greater than or equal to 92%, while only the Turning activity shows about 91.77% accuracy in the E2 scenario. There is some misclassification between the No movement and Sitting/Standing activities for both E1 and E2 scenarios. The possible reason could be that the signal pattern between the two activities is similar.

**Figure 9:**
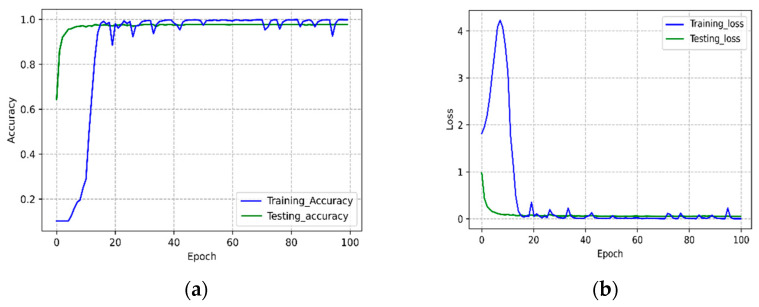
Accuracy (**a**) and Loss (**b**) graph of the STC-NLSTMNet model.

The accuracy and loss history graphs of the STC-NLSTMNet model are displayed in Figure 9. The training and testing graph (Figure 9a) of the STC-NLSTMNet model converges quite quickly within 100 epochs. It is rational for the training loss to be relatively higher than the testing loss, as shown in Figure 9b. This is because the training involves going through numerous phases to learn the various CSI signal patterns among different activities.

**Figure 10:**
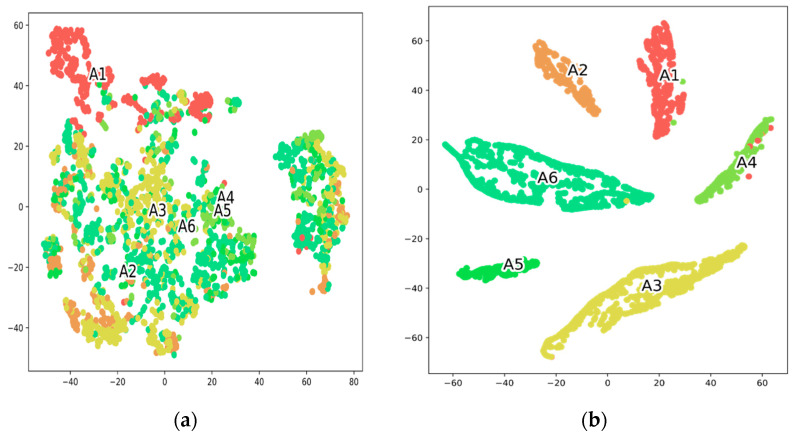
Two-dimensional T-SNE visualization of the testing data before (**a**) and after (**b**) learning representations of the proposed STC-NLSTMNet model.

T-SNE plots help assess the generalization ability of any model and how well the model reflects data in a large-scale feature dimension. Figure 10b illustrates the application effect of T-SNE where samples are well distributed and samples of the same class are grouped together. On the contrary, Figure 10a depicts the sample before applying T-SNE, where the samples are cluttered and difficult to identify. The clean and well-defined separation between all activities exhibited the capability of the proposed STC-NLSTMNet to partition the feature dimension.

### 6.3. Performance Comparison

Since the dataset plays an important role in a model’s overall evaluation, it is rational to compare various models using the same dataset. As a result, we evaluate how well the proposed system performs in comparison to other systems using the same StanWiFi and Multi-environment datasets, which are shown in Table 7. Yousefi, et al. [41] presented a survey of human action recognition. Six different activities from six different people were gathered. They used RF, HMM, and LSTM to validate the dataset and stated that LSTM produced the best result, with a score of 90.5%. Chen, et al. [19] proposed attention-based bi-directional LSTM (ABLSTM). They used attention to obtain the more essential features. Their proposed ABLSTM model provided 97.3% accuracy on the StanWiFi dataset. Yadav, et al. [42] proposed a modified InceptionTime model named CSITime and provided an accuracy of 98%, F1-score of 99.01%, precision of 99.16%, and recall of 98.87% on the StanWiFi dataset. Salehinejad, et al. [54] presented a DL-based lightweight human activity recognition called the LiteHAR model. Without training, they extracted features using a convolutional kernel, and for the final classification, they utilized a Ridge regression classifier. Their LiteHAR model achieved 93% accuracy on the StanWiFi dataset. Whereas, our work presented the best recognition result compared to other existing models, as presented in Table 7, with an average accuracy of 99.88%, precision of 99.72%, recall of 99.73%, and F1-score of 99.72% using the STC-NLSTMNet model on the same dataset. Our proposed STC-NLSTMNet increases the accuracy by 1.88% than existing state-of-the-art [42] on the same StanWiFi dataset.

In the case of the Multi-environment dataset, Alsaify, et al. [49] used only the LOS scenario collection dataset (office room and hall room) to validate their model. They initially filtered the signal and eliminated outliers. Then, relevant features were retrieved, and necessary features were chosen. Using the chosen features, an SVM classifier was trained, and the accuracy rate was 91.27% regarding the office room (E1) scenario and 86.53% regarding the hall room (E2) scenario, respectively. Alsaify, et al. [55] used three signal processing techniques and extracted time and frequency domain features. After that, they selected the best subsets of features and the selected subsets were trained using SVM and achieved 94% and 89.07% accuracy for the office room (E1) and hall room (E2), respectively. At the same time, our proposed STC-NLSTMNet achieves 98.20% and 96.65% accuracy in terms of the E1 and E2 scenario on the Multi-environment dataset, which is 4% and 7.6% higher than the existing best work [55], respectively. To our knowledge, no other researchers have worked with Multi-environment datasets acquired in an NLOS (E3) environment. Our model shows 94.68% accuracy with a precision of 94.57%, recall of 94.55%, and F1-score of 94.56% for the E3 scenario. Observing the performance of other existing works in Table 7, it can be seen that our proposed STC-NLSTMNet model performs the best on both datasets. This improvement could be the newly proposed architecture (STC-NLSTMNet), which can learn and utilize both spatial and temporal features, and optimal selection of hyperparameters.

## 7. Conclusions

A DL-based model called STC-NLSTMNet is proposed to automatically recognize human activities from WiFi CSI signals. We have seen WiFi CSI-based human activity is time-series data with spatial and temporal features, and proposed an STC-NLSTMNet model capable of extracting both features simultaneously. The proposed model utilizes DS-Conv blocks for extracting spatial features and FAM to pay attention to the most significant features. Additionally, the NLSTM enhances the depth of LSTM through nesting to efficiently capture the hidden intrinsic temporal features in CSI signals. Thus, the combination of DS-Conv, FAM, and NLSTM enhances the model’s capabilities to represent both features in the CSI signal and to better focus attention on activity information; therefore, the activity recognition accuracy of the STC-NLSTMNet model is improved. The performance of the proposed STC-NLSTMNet model has been evaluated on two datasets, and achieved activity recognition accuracies of 99.88% and 98.20% for the StanWiFi and Multi-environment, respectively. In addition, we have provided a comparative analysis of the results with other existing work, and our proposed STC-NLSTMNet model shows 1.88% and 4% higher accuracies, respectively, compared to the best existing works. Although our proposed STC-NLSTMNet model shows better recognition accuracy on the LOS environment regarding both datasets, its shows comparatively low performance in the NLOS environment, which needs further investigation.

In real life, recognizing multi-user activity is a more realistic and more challenging scenario than recognizing single-user activity. In the future, we will extend the work proposed in this study to the problem of multi-user human activity recognition. The publicly available datasets have common activities that are beyond the real scenario, as an average individual performs numerous activities daily. Therefore, collecting a dataset using WiFi that contains other daily human activities in an indoor environment is left for another future work.

## Figures and Tables

**Table 1:** Summary of Multi-environment dataset.

Activity Label	Activity Name	Details	Number of Samples
A1	No movement	Standing still or sitting still or laying down	7513
A2	Falling	Falling from a sitting position or standing position	2405
A3	Walking	Walking to the transmitter or to the receiver	2408
A4	Sitting/Standing	Sitting on a chair or standing from a chair	1612
A5	Turning	Turning to the receiver or to the transmitter	2412
A6	Pick up	Picking up a pen from the ground	1421

**Table 2:** Summary of the StanWiFi dataset.

Activity Name	Number of Samples	Activity Name	Number of Samples
Walk	4707	Sit down	1394
Fall	1510	Lay down	2175
Stand up	1057	Run	3889

**Table 3:** Summary of the STC-NLSTMNet model.

Section	Layer Type	Output Shape	Parameters
Spatial-temporal feature extraction	Conv 2D	256 × 45 × 32	320
	BN and ReLU	256 × 45 × 32	128
	DS-Conv block	128 × 23 × 64	2816
	FAM	128 × 23 × 64	49
	DS-Conv block	64 × 12 × 128	9728
	FAM	64 × 12 × 128	49
	DS-Conv block	32 × 6 × 512	69,888
	GAP	1 × 512	0
	NLSTM (50)	512 × 50	30,600
	TDFC (20)	512 × 20	1020
Recognition	GAP	1 × 20	0
	FC(20)	1 × 20	420
	Softmax	1 × 6	126

**Table 4:** Performance result of the proposed STC-NLSTMNet model on the StanWiFi dataset.

Scenario’s	Metrics	Fold										Average
		1st	2nd	3rd	4th	5th	6th	7th	8th	9th	10th	
StanWiFi	Accuracy	99.86	99.86	99.93	99.89	99.83	99.86	100	99.89	99.90	99.80	99.88
	Precision	99.66	99.81	99.9	99.85	99.79	98.99	99.82	99.79	99.85	99.73	99.72
	Recall	99.79	99.76	99.86	99.81	99.8	99.25	99.75	99.81	99.79	99.68	99.73
	F1-score	99.72	99.78	99.88	99.83	99.79	99.12	99.78	99.80	99.82	99.71	99.72

**Table 5:** The results of the STC-NLSTMNet model’s performance on the Multi-environment dataset on LOS.

Scenario’s	Metrics	Fold										Average
		1st	2nd	3rd	4th	5th	6th	7th	8th	9th	10th	
E1	Accuracy	98.49	98.32	97.93	98.21	97.93	98.17	97.99	98.35	97.96	98.67	98.20
	Precision	98.35	98.29	97.79	98.05	97.86	98.13	97.87	98.31	97.74	98.62	98.10
	Recall	98.39	98.25	97.84	98.09	97.85	98.03	97.86	98.28	97.71	98.58	98.08
	F1-score	98.37	98.27	97.82	98.07	97.86	98.08	97.87	98.30	97.73	98.60	98.09
E2	Accuracy	96.47	96.86	96.82	95.91	96.86	96.04	97.04	97.04	96.91	96.56	96.65
	Precision	96.44	96.71	96.76	95.82	96.79	95.93	97.00	96.94	96.69	96.36	96.54
	Recall	96.41	96.73	96.72	95.87	96.36	95.78	95.95	97.01	96.85	96.46	96.41
	F1-score	96.43	96.72	96.74	95.85	96.57	95.85	96.47	96.97	96.77	96.41	96.48

**Table 6:** The results of the STC-NLSTMNet model’s performance on the Multi-environment dataset on NLOS.

Scenario’s	Metrics	Fold										Average
		1st	2nd	3rd	4th	5th	6th	7th	8th	9th	10th	
E3	Accuracy	94.68	94.28	94.00	94.73	93.83	95.00	94.28	95.63	95.29	95.12	94.68
	Precision	94.53	94.19	93.92	94.65	93.79	94.89	94.13	95.45	95.13	95.02	94.57
	Recall	94.54	94.21	93.88	94.55	93.75	94.76	94.11	95.45	95.23	95.01	94.55
	F1-score	94.53	94.2	93.9	94.60	93.77	94.82	94.12	95.45	95.18	95.02	94.56

**Table 7:** Comparison between the proposed system and other existing work.

Dataset	Study	Environment	Method and Year	Metrics (%)			
				Accuracy	Precision	Recall	F1-Score
StanWiFi	Yousefi et al. [41]	LOS	LSTM (2017)	90.5	---	---	---
	Chen et al. [19]		ABLSTM (2018)	97.3	---	---	---
	Santosh et al. [42]		CSITime (2022)	98.00	99.16	98.87	99.01
	Shahrokh et al. [54]		LiteHAR (2022)	93.00	---	---	---
	**proposed**		**STC-NLSTMNet**	**99.88**	**99.72**	**99.73**	**99.872**
Multi-environment	Alsaify et al. [49]	LOS (E1)	SVM (2022)	91.27	---	---	---
	Alsaify et al. [55]		SVM (2021)	94.03	---	---	---
	**proposed**		**STC-NLSTMNet**	**98.20**	**98.10**	**98.08**	**98.09**
	Alsaify et al. [49]	LOS (E2)	SVM (2022)	86.53	---	---	---
	Alsaify et al. [55]		SVM (2021)	89.07	---	---	---
	**proposed**		**STC-NLSTMNet**	**96.65**	**96.54**	**96.41**	**96.48**

## Data Availability

The dataset used in this study is available at the following website: https://data.mendeley.com/datasets/v38wjmz6f6/1 (accessed on 16 November 2022).
